# Neuroblastoma—A Neural Crest Derived Embryonal Malignancy

**DOI:** 10.3389/fnmol.2019.00009

**Published:** 2019-01-29

**Authors:** John Inge Johnsen, Cecilia Dyberg, Malin Wickström

**Affiliations:** Childhood Cancer Research Unit, Department of Women’s and Children’s Health, Karolinska Institutet (KI), Stockholm, Sweden

**Keywords:** neuroblastoma, neural crest, tumorigenesis, oncogenic drivers, mutation

## Abstract

Neuroblastoma is a neural crest derived malignancy of the peripheral nervous system and is the most common and deadliest tumor of infancy. It is characterized by clinical heterogeneity with a disease spectrum ranging from spontaneous regression without any medical intervention to treatment resistant tumors with metastatic spread and poor patient survival. The events that lead to the development of neuroblastoma from the neural crest have not been fully elucidated. Here we discuss factors and processes within the neural crest that when dysregulated have the potential to be initiators or drivers of neuroblastoma development. A more precise biological understanding of neuroblastoma causes and cell of origin is highly warranted. This will give valuable information for the development of medicines that specifically target molecules within neuroblastoma cells and also give hint about the mechanisms behind treatment resistance that is frequently seen in neuroblastoma.

## Introduction

Neuroblastoma is a malignancy of the sympathetic nervous system that almost exclusively occurs in early childhood. Neuroblastoma is a relatively rare disease affecting 1 in 8,000 live births and represents 6%–10% of all childhood tumors. However, neuroblastoma accounts for 12%–15% of all childhood cancer related death and is the most common and deadly extracranial tumor of childhood (Brodeur, 2003; Johnsen et al., 2009; Maris, 2010; Park et al., 2010). The median age at diagnosis for neuroblastoma is 17–18 months and approximately 40% of the patients are younger than 1 year at diagnosis whereas less than 5% of the patients are older than 10 years (London et al., 2005; Maris, 2010; Park et al., 2010).

Histopathologically, neuroblastoma has been defined as a small blue round cell tumor of childhood, a group of diagnoses consisting mostly of undifferentiated pediatric malignancies. The cell of origin for neuroblastoma has yet to be precisely defined but probably derives from sympathoadrenal progenitor cells within the neural crest that differentiate to sympathetic ganglion cells and adrenal catecholamine-secreting chromaffin cells (L’Abbate et al., 2014). Neuroblastoma manifest anywhere along the sympathetic nervous system, the majority are located in the abdomen along the sympathetic chain and in the adrenal gland medullary region. Neuroblastoma is characterized by wide clinical heterogeneity which includes everything between spontaneous regression or differentiation and treatment-refractory progression despite intensive therapy. This heterogeneity is correspondingly reflected in the survival of neuroblastoma patients spanning from 85 to 90% cure rate in patients with low- and intermediate-risk disease to less than 50% in patients diagnosed with high-risk neuroblastoma (Pearson et al., 2008; Matthay et al., 2009; Valteau-Couanet et al., 2014; Ladenstein et al., 2017).

Since the majority of neuroblastoma patients are diagnosed with high-risk disease in which current intensive therapies are not effective, the development of new treatment options for this group of patients as well as for those patients with relapsed or recurrent tumors, where the current overall survival is less than 10%, is urgently needed. To accomplish this there is also a need for a more thorough and precise knowledge of the molecular landscape of neuroblastoma cells and their interactions with the surrounding microenvironment.

## Molecular Pathogenesis of Neuroblastoma

A number of biological and genetic markers of neuroblastoma have been investigated in order to facilitate diagnosis, prognosis and monitoring of treatment effects in patients with neuroblastoma (Riley et al., 2004). However, probably due to the complexity of the disease, no single biological or genetically marker has proven useful for accurate diagnosis. Instead a combination of imaging techniques, measurement of secreted catecholamine metabolites, cell surface markers and chromosomal and genetic analysis of tumor DNA are used for diagnosis and risk classification in patients with neuroblastoma.

### Cell Surface Markers in Neuroblastoma

One surface antigen that is abundantly present in the outer membrane of all neuroblastomas regardless of disease stage is disialoganglioside (GD2). During embryonal development, GD2 is expressed on neural and mesenchymal stem cells, whereas postnatal expression of GD2 is in normal tissues restricted to neural cells located in the peripheral and central nervous system as well as in melanocytes of the skin (Lammie et al., 1993; Yanagisawa et al., 2011). The detailed function of GD2 during normal development is currently not understood. However, gangliosides, including GD2 have been proposed to be important for the formation and maintenance of membrane microdomains in neural tissues (Ohmi et al., 2012). Although immunohistochemical detection of GD2 in tumor tissue samples can be used as an indicator of neuroblastoma, the most valuable consequence of GD2 expression has been the utilization of GD2 as a neoantigen for immunotherapy. Anti-GD2 antibodies have for the last 20 years been successfully applied as a treatment option for high-risk neuroblastoma and recent studies have shown further increased survival of patients with high-risk neuroblastoma treated with a combination of chimeric anti-GD2 antibodies, GM-CSF and IL-2 suggesting more systematic evaluation of this treatment regimen (Yu et al., 2010; Ladenstein et al., 2013; Mody et al., 2017).

The neurotrophin receptors, TrkA, TrkB and TrkC are receptor tyrosine kinases that each is crucial for proper development and maintenance of the peripheral nervous system (Brodeur, 2018). Numerous studies have shown that TrkA and TrkB have important implications for the diverse pathology observed in neuroblastoma patients (Brodeur et al., 1997, 2009; Thiele et al., 2009). High expression of TrkA is a hallmark for low-grade neuroblastomas prone to spontaneous regression or differentiation whereas high expression of TrkB has been linked to high-risk disease and poor patient survival (Kogner et al., 1993; Nakagawara et al., 1993, 1994). In addition, high-risk neuroblastoma also expresses the TrkB ligand brain-derived neurotrophic factor (BNDF) resulting autocrine/paracrine tumor cell survival (Acheson et al., 1995; Matsumoto et al., 1995).

### Chromosomal and Genetic Markers in Neuroblastoma

Pediatric cancers harbor substantial less genomic aberrations and mutations compared to adult tumors (Cheung et al., 2012; Molenaar et al., 2012; Pugh et al., 2013). Low-risk neuroblastoma often present with whole chromosomal gains and the tumor cells are frequently hyperdiploid (near triploid or penta/hexaploid) in their chromosomal make-up (Ambros et al., 2009). On the contrary, high-risk neuroblastoma contains segmental chromosomal aberrations that affect only a part of a given chromosome (Carén et al., 2010; Morgenstern et al., 2014; Irwin and Park, 2015; Matthay et al., 2016). The most frequent chromosomal aberration associated with poor prognosis in neuroblastoma is somatically acquired amplification of the *MYCN* gene, hemizygous deletions of 1p and 11q, and segmental gain of 17q (Brodeur, 2003; Maris, 2010). In addition, high-risk neuroblastomas can also display genomic rearrangements at chromosomal region 5p15.33 which is located proximal of the telomerase reverse transcriptase gene (*TERT*) resulting in chromosomal changes, DNA methylation and enhanced TERT expression (Peifer et al., 2015; Valentijn et al., 2015).

Genomic surveys using exome- and whole-genome sequencing of neuroblastoma tissue samples showed a low somatic mutation count (12–18, median 15) and that there is no single genetic mutation event that can explain the development of all neuroblastoma cases (Cheung et al., 2012; Molenaar et al., 2012; Pugh et al., 2013). The *anaplastic lymphoma kinase* (*ALK)* is the most frequent mutated gene detected in 7%–10% primary neuroblastomas. *ALK* mutations are also present in almost all cases of familial neuroblastomas which account for 1%–2% of the neuroblastoma cases (Mossé et al., 2008). Germline loss-of-function mutations of the *paired-like homeobox 2B* (*PHOX2B*) gene has also been reported in familial neuroblastoma as well as in approximately 4% of high-risk spontaneous neuroblastoma cases (Mossé et al., 2008; Cheung et al., 2012; Pugh et al., 2013). In addition, somatic mutations in primary neuroblastomas has been reported for the transcriptional regulator *ATRX* (2.5% inactivating mutations), the tumor suppressor p53 (*TP53*; 1%–2% in primary tumors, 10% in recurrent and relapsed tumors) gene, *ARID1A/1B* (2%–3% inactivating mutations), *PTPN11* (2.9% activating mutations), *MYCN* (1.7% activating mutations), *NRAS* (0.83% activating mutations) and *BRCA2* (Cheung et al., 2012; Molenaar et al., 2012; Pugh et al., 2013; Brodeur and Bagatell, 2014). However, the importance and relevance of several of these mutations for neuroblastoma development have yet to be identified. Other less frequently detected mutations in primary neuroblastomas are often in genes in which the protein is involved in the regulation of signal transduction pathways as exemplified by detection of genetic lesions in the MAPK signaling cascade in 3%–5% of primary neuroblastomas (Pugh et al., 2013) and in 28% of genes responsible for correct neuritogenesis, many in the Rho family of genes (Molenaar et al., 2012; Dyberg et al., 2017). More recent data indicate an accumulation of gene mutations in recurrent and relapsed neuroblastomas and the presence of frequent inter- and intra-tumorigenic genetic heterogeneity in individual patients which further add complexity to the molecular pathogenesis of the disease (Schleiermacher et al., 2014; Eleveld et al., 2015; Schramm et al., 2015; Braekeveldt et al., 2018).

Genome-wide association studies (GWAS) further add to the heterogeneity of the genetic landscape observed in neuroblastoma since it has been shown that there are at least a dozen highly significant polymorphic alleles that can influence the formation of neuroblastoma (Bosse and Maris, 2016). Although, each association has modest individual effect on disease initiation, multiple associations can cooperate within a patient to promote tumorigenesis. Some of the GWAS-defined neuroblastoma susceptibility genes which include *CASC15*, BRCA1-associated RING domain protein 1 (*BARD1*), *LMO1*, *DUSP12*, *DDX4*, *IL31RA*, *HSD17B12*, *HACE1*, *LIN28B*, and *NEFL* have been shown to display oncogenic or tumor suppressive functions in established disease (Maris et al., 2008; Capasso et al., 2009, 2013, 2014; Diskin et al., 2009; Wang et al., 2011; Bosse et al., 2012; Pandey et al., 2014; Oldridge et al., 2015; Russell et al., 2015). Polymorphic alleles within the *lin-28 homolog B* (*LIN28B*) locus have been linked to neuroblastoma development and deregulated expression of *LIN28B* induces expression of MycN (Diskin et al., 2012; Molenaar et al., 2012). Lin28B also regulates microRNA (miRNA) biogenesis through depletion of the Let-7 family of miRNAs and modulate the activity of the nuclear GTP-binding protein RAN and the stability of Aurora A kinase (*AURKA*; Schnepp et al., 2015). This suggests that Lin28B-Ran-Aurora A kinase signaling can drive neuroblastoma tumorigenesis and that this pathway could be used as a therapeutic target (Matthay et al., 2016). Another polymorphic locus that has been strongly associated with neuroblastoma and poor prognosis is *BARD1* located at chromosome 2q35. Fine mapping of *BARD1* using GWAS identified a variant located in the canonical promoter region of *BARD1* altering the binding site of the transcription factor HSF1 and correlated with low expression of full-length Bard1. Low expression of Bard1 induces proliferation and invasion of neuroblastoma cells indicating that Bard1 has a tumor suppressor function in neuroblastoma (Cimmino et al., 2018).

GWAS has also identified a locus at chromosome 6p22.3 harboring single nucleotide polymorphisms (SNPs) associated with increased risk of neuroblastoma in particular high-risk neuroblastomas (Maris et al., 2008; Russell et al., 2015). These SNPs at the 6p22.3 locus are located in the intron regions of a long noncoding RNA (lncRNA) gene *CASC15* and in *NBAT1* encoding a lncRNA-transcribed antisense to *CASC15* (Pandey et al., 2014). Recent data shows that *CASC15* and *NBAT1* promote differentiation by interactions with *SOX9* and *USP36*, located on chromosome 17q that is frequently gained in high-risk neuroblastomas. Lack of these lncRNAs at 6p22.3 could lead to accumulation of undifferentiated cells within the neural crest and tumorigenesis (Mondal et al., 2018).

Taken together the molecular heterogeneity observed in neuroblastoma represent clinical challenges since tumors that seemingly are phenotypically and morphological very similar will have completely different responses to treatment depending on the molecular landscape of the tumor. This proposes that neuroblastoma patients in general and specifically those belonging to the high-risk groups should undergo careful examination to elucidate the molecular make-up of the tumor and that the treatments are designed to target the molecular aberrations detected in the individual tumor samples as a supplement to the current conventional therapies (Johnsen et al., 2018).

## Candidate Factors Within the Neural Crest Linked to Neuroblastoma Development

Although many molecular prognostic factors have been described in neuroblastoma only activating *ALK* mutations and MYCN overexpression have been proven to be *de novo* oncogenic drivers as mutation or overexpression of these molecules give rise to neuroblastoma in genetically engineered mouse models (Weiss et al., 1997; Heukamp et al., 2012). Mice with targeted expression of *Lin28b* to sympathetic adrenergic lineage cells also develop neuroblastoma. However, the tumor development in this mouse model is induced by Lin28B-mediated downregulation of Let-7 resulting in overexpression of MycN protein (Molenaar et al., 2012).

### MYCN as an Oncogenic Driver in Neuroblastoma

**Figure 1:**
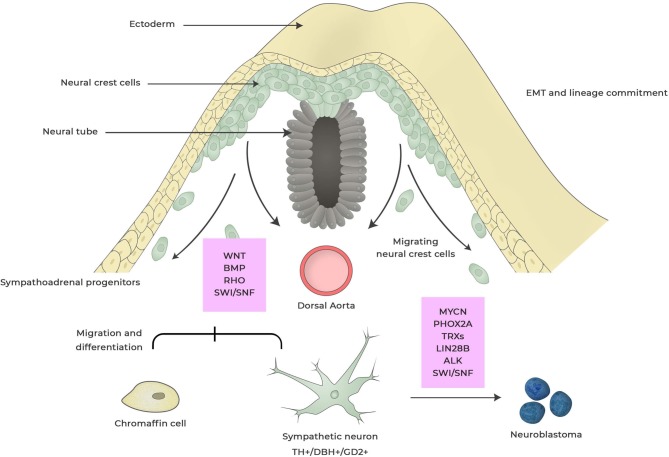
Neuroblastoma development from neural crest. During embryogenesis cells within the neural crest undergo epithelial-to-mesenchymal transition (EMT) enabling cells to delaminate, migrate and differentiate into a wide range of cell types that contributes to anatomical structures within the organism. This process is regulated by a complex set of external signaling, activation of transcriptional programs and epigenetic events. Dysregulation of factors involved in this process can induce changes in cell specification and deregulated-migration and -cell differentiation giving rise to hyperneoplastic lesions that eventually can result in neuroblastoma. These events can occur during different stages of neural crest maturation and factors that are thought to be important for neuroblastoma development are boxed in red. Dysregulated expression of MYCN is the most powerful oncogenic driver for neuroblastoma and induces proliferation and inhibits apoptosis of sympathoadrenal lineage cells whereas *LIN28B* control MYCN expression via regulating Let-7 miRNA. *Anaplastic lymphoma kinase (ALK)* and *paired-like homeobox 2B (PHOX2B)* are germline mutations found in neuroblastoma. Rho signaling is crucial for the migration of neural crest cells by controlling contact inhibition of locomotion between neural crest cells. SWI/SNF complexes are tumor suppressors that for coordinate diverse chromatin alterations which influences the transcriptional output, DNA replication and repair.

High expression of MycN is detected in the early post-migratory neural crest (Figure 1) and is important for regulation of ventral migration and expansion of cells within the neural crest during normal murine sympathoadrenal development (Zimmerman et al., 1986). In differentiating sympathetic neurons, the levels of MycN is gradually reduced suggesting that sympathoadrenal maturation is independent of MycN expression (Zimmerman et al., 1986; Wartiovaara et al., 2002; Hansford et al., 2004). Sympathoadrenal precursor cells maturates into neural or chromaffin cells and it is thought that preneoplastic lesions that can develop into neuroblastoma occur in sympathoadrenal precursor cells that not have received or responded to signals that determine the neuronal or chromaffin cell fate (Zimmerman et al., 1986; Marshall et al., 2014). Studies in zebrafish have shown that forced MycN expression in sympathoadrenal precursor cells blocks the development of chromaffin cells leading to the development of neuroblastoma (Zhu et al., 2012). During sympathoadrenal development an excess of precursor cells are produced that during the final normal maturation stages undergo controlled apoptosis caused by local neural growth factor deprivation (Yuan and Yankner, 2000). MycN being a master transcription factor is involved in both cell proliferation and apoptosis (Rickman et al., 2018). Hence, persistent MycN expression during the maturation stages of sympathoadrenal precursors could result in blockage of apoptotic signaling and sustained proliferation eventually resulting in neuroblastoma development.

Transgenic mice where the human *MYCN* cDNA is placed in front of the tyrosine hydroxylase promoter (*Th-MYCN*) spontaneously develop neuroblastomas which are morphological and phenotypical similar to human high-risk neuroblastomas (Weiss et al., 1997; Rasmuson et al., 2012). Neuroblastoma development in this mouse model is dependent on gene dosage. All *Th-MYCN* mice that are homozygous (*MYCN*^+/+^) will develop tumors 4.0–6.9 weeks of age whereas 50% of the hemizygous mice (*MYCN*^+/−^) develop tumors at 5.6–19 weeks of age (Rasmuson et al., 2012). More recent, a refined genetically engineered mouse model with *Cre*-conditional induction of *MYCN* in dopamine β-hydroxylase-expressing cells termed *LSL-MYCN;Dbh-iCre* was shown to give rise to clinically relevant neuroblastomas in 75% of the mice (Althoff et al., 2015). Furthermore, overexpression of *MYCN* in primary neural crest cells result in neuroblastoma formation when these cells are transduced back into mice (Olsen et al., 2017). Also, ectopic expression of *MYCN* in the neural crest of zebrafish results in neuroblastoma development (Zhu et al., 2012). Together these data strongly suggest that increased and sustained expression of MycN in cells within the neural crest is sufficient for neuroblastoma development and establish *MYCN* as a major oncogenic driver in neuroblastoma.

### LIN28B as an Inducer of Neuroblastoma

The RNA binding proteins Lin28B specifically target Let-7 miRNAs by binding to the terminal loop or the pre-element region of pre-miRNAs (Newman et al., 2008; Rybak et al., 2008; Viswanathan et al., 2008). This prevents Drosha-mediated cleavage and maturation of the Let-7 pre-miRNA by the microprocessor complex (Piskounova et al., 2011). During embryogenesis Lin28B regulates the expression of Let-7 and control the developmental timing and proliferation during neural crest linage commitment (Figure 1; Rybak et al., 2008). Lin28B has also been shown to be important for the maintenance of stemness during embryonic development (Yu et al., 2007). Together these functions of Lin28B in the neural crest may result in maintenance of an undifferentiated phenotype (Rybak et al., 2008; Molenaar et al., 2012; Marshall et al., 2014). Lin28B-mediated downregulation of Let-7 miRNAs increases MycN expression and targeted ectopic expression of Lin28B in mouse neural crest cells results in MycN-driven neuroblastoma (Molenaar et al., 2012). Lin28B indirect regulation of MycN expression by downregulation of Let-7 pre-miRNAs correspond well with a model that neuroblastoma is initiated by aberrant regulation of key developmental proteins (Marshall et al., 2014).

### ALK Mutations as Accelerators of Neuroblastoma Development

More than 35 different mutations in *ALK* have been reported in neuroblastoma (Hallberg and Palmer, 2016). The majority of the mutations are point mutations most frequently detected in one of three hotspot residues in the kinase domain: F1174, F1245 and R1275 resulting in Alk activation (Bresler et al., 2014; Hallberg and Palmer, 2016). During mouse embryogenesis Alk is highly expressed in the developing nervous system, a pattern which is recapitulated in chicken where high expression of Alk is found in a subset of spinal motor neurons, the sympathetic ganglia and dorsal root ganglia (Figure 1; Iwahara et al., 1997; Hurley et al., 2006; Vernersson et al., 2006; Palmer et al., 2009). Enhanced proliferation of sympathetic neurons and enlargement of the sympathetic ganglion is evident in mice expressing mutant *Alk* (Cazes et al., 2014). ALK has also been shown to signal trough Midkine (neurite growth promoting factor-2; NEGF-2) which could be important for proliferation of the sympathoadrenal lineage during development (Reiff et al., 2011). This together with the fact that Alk mediates signaling via the JAK/STAT, RAS/MAPK, PI3K and PLC*γ* pathways (Palmer et al., 2009), some of which have important functions during the development of the peripheral nervous system suggest that aberrant Alk activation plays a major role in processes leading to cellular transformation within the neural crest.

Gain-of-function mutation of ALK^F1174F^ can induce neuroblastoma in mice if ALK^F1174F^ is under control of the dopamine-β-hydroxylase (*Dbh*) promoter but require high Mycn expression in order to develop neuroblastoma when ALK^F1174F^ expression is controlled by the *Th* promoter in both mice and zebrafish (Weiss et al., 1997; Berry et al., 2012; Zhu et al., 2012). Also, the tumor penetrance in *dbh*: ALK^F1174F^ mice is less efficient with 40% of the mice having neuroblastoma at 50 weeks of age, compared to *Th-MYCN* transgenic mice (Weiss et al., 1997; Rasmuson et al., 2012). The reasons for this discrepancy in tumor penetrance between *Th*- and *Dbh*-ALK^F1174F^ mice are not currently understood (Matthay et al., 2016). Also, *Alk*^*R1279Q*^ and *Alk*^*F1178L*^ knock-in mouse models did not develop tumors but both *Alk* mutations accelerated neuroblastoma development and increased the tumor penetrance when *Alk*^*R1279Q*^ or *Alk*^*F1178L*^ knock-in mice were crossed with *Th-MYCN* mice (Cazes et al., 2014). Together these results suggest that *ALK* is an accelerator and require other genetic insults in order to develop neuroblastoma.

## Potential Factors for Neuroblastoma Tumorigenesis

The recent development of high-throughput techniques for analyzing genomic DNA, RNA and epigenetic profiling have given valuable information on the molecular landscape of neuroblastoma and guidance to factors that are important for the initiation, development and progression of neuroblastoma from cells within the neural crest.

### Mutations of Genes Involved in the Epithelial-to-Mesenchymal Transition During the Migration of Neural Crest Cells as Potential Factors for Neuroblastoma

A coordinated set of signals mainly derived from Wingless (Wnt), bone morphogenetic protein (BMP) and fibroblast growth factor (FGF) are required to initiate migration of neural crest cells by acquiring cell motility through epithelial–mesenchymal transition (EMT, Figure 1; Goldstein et al., 2005). The non-canonical Wnt-planar cell polarity (PCP) signaling cascade is fundamental for the mobilization and initiation of migration of neural crest cells by controlling contact inhibition of locomotion between cells within the neural crest. PCP proteins are controlling the activity of Rho GTPases locally by activating or inhibiting RhoA and Rac1, resulting in cells migrating away from each other upon collision (Figure 1; Sebbagh and Borg, 2014). The activation of Rho signaling results in the activation of downstream serine/threonine kinases called Rho-Associated Coiled-Coil Containing Protein Kinases, ROCK1 and ROCK2 (Anastas and Moon, 2012). ROCK1 and ROCK2 phosphorylate downstream substrates, mainly myosin light chain (MLC) and LIMK1/2, which control several cellular functions predominately through rearrangement of the actin cytoskeleton (Riento and Ridley, 2003; Hahmann and Schroeter, 2010). Genes associated with Rho/Rac signaling are frequently mutated in neuroblastoma and high ROCK2 expression correspond to poor prognosis (Molenaar et al., 2012; Dyberg et al., 2017). Also, mutations of Rho GTPase genes have been detected in low frequencies in various other cancers (Alan and Lundquist, 2013). Interestingly, recurrent somatic oncogenic driver mutations of Rac1 have been identified in 5%–9% of tumor DNA from patients with melanoma which similar to neuroblastoma also originates from cells within the neural crest (Berger et al., 2012; Krauthammer et al., 2012). This suggests that the mutations of Rho GTPases and related genes seen in neuroblastoma and melanoma are obtained during migration and differentiation of neural crest cells. The importance of the different mutations of Rho GTPases and associated genes in neuroblastoma is not known. The majority of these mutations have the potential to activate RhoA, inhibit Rac1 (Molenaar et al., 2012) and increase the activity of ROCK proteins (Dyberg et al., 2017). As Rho signaling is highly activated during the initiation and migration of neural crest cells, deregulated Rho/Rac1 signaling could represent an oncogenic hit during neuroblastoma development. Also, RhoA, Rac1 and Cdc42 have been implicated in both Eph/ephrin internalization and signaling. Eph/ephrin signaling plays essential roles during migration of neural crest cells, such as cytoskeleton remodeling and axon guidance but also stimulate angiogenesis and tissue separation (Baker and Antin, 2003; Xu and Wilkinson, 2013; Wislet et al., 2018). High expression levels of Ephb6 and its ligand ephrin-B2 and ephrin-B3 is associated with low-stage neuroblastoma (Tang et al., 2000). Finally, high expression of the basic-loop-helix transcription factor NeuroD1 is associated with poor prognosis in patients with neuroblastoma and NeuroD1 was found to be highly expressed in hyperplastic regions consisting of neuroblasts in the celiac sympathetic ganglion of 2 weeks old *Th-MycN* mice developing neuroblastoma (Huang et al., 2011). Knockdown experiments using shRNA directed against NeuroD1 inhibited the motility of neuroblastoma cells which was associated with induction of Slit2 expression (Huang et al., 2011). Slit proteins (Slit1-Slit3) are extracellular matrix glycoproteins that upon binding to the repulsive guidance receptor Roundabout (Robo1-Robo4) family regulate migration of neural crest cells (Wong et al., 2001). The Slit–Robo GTPase-activating proteins (srGAPs) are Slit-Robo effector proteins that regulate the activity of RhoGTPase family members and are important for proper migration of neural crest cells (Wong et al., 2001; Blockus and Chédotal, 2016).

### Mutations of Genes Guiding Neural Crest Lineage Commitment as Inducers of Neuroblastoma

During embryogenesis the differentiation of sympathetic neurons in the neural crest is induced by members of the family of BMPs such as BMP-2, BMP-4 and BMP-7 (Figure 1) expressed by smooth muscle cells of the dorsal aorta (Varley et al., 1995; Reissmann et al., 1996; Shah et al., 1996; Varley and Maxwell, 1996). BMPs induce a differentiation in sympathetic neural precursors by controlling a set of transcription factors including Phox2b, Phox2a, Ascl1, Insm1, Hand2, Gata2/3, Sox4 and Sox11 (Howard et al., 2000; Tsarovina et al., 2004; Rohrer, 2011). In this transcriptional network *PHOX2B* (Figure 1) has been identified as a master gene of autonomic neuron development since *Phox2b* knockout in mice results in the absence of the entire peripheral autonomic nervous system (Pattyn et al., 1999). Heterozygous germline mutations as well as somatic mutations of *PHOX2B* are present in a subset of neuroblastomas. These mutations are often clinically presented in combination with the neural crest disorders Hirschsprung disease and congenital hypoventilation syndrome highlighting the connection between neuroblastoma and defective neural crest development. Mutation within *PHOX2B* blocks the transcriptional network responsible for the differentiation of sympathetic neurons and allows continuous proliferation of neural precursors cells (Pattyn et al., 1999; Reiff et al., 2010) which potentially could result in the development of pre-neoplastic lesions within the developing neural crest.

### Chromatin Remodeling Molecules as Factors for Neuroblastoma Development

Sporadic *α-thalassaemia/mental retardation syndrome X-linked* (*ATRX*) mutations have been detected in a subset of neuroblastoma patients mainly in children older than 18 months having poor prognosis (Cheung et al., 2012). *ATRX* encodes a SWI/SNF chromatin-remodeling ATP-dependent helicase (Figure 1) that is included in ATP-dependent chromatin remodeling complexes which are responsible for reorganization of the nucleosome to make DNA accessible during transcription, replication and DNA repair (Tang et al., 2010). *ATRX* mutations are prevalent in X-linked mental retardation (XLMR) and α-thalassemia syndromes. However, no increase in neuroblastoma incidence has been observed in children with XLMR suggesting that ATRX mutations alone are not sufficient to induce neoplasia (Cheung and Dyer, 2013). Atrx being a part of SWI/SNF chromosomal remodeling complexes which are important for guiding transcription and genome integrity as well as being associated to the recombination based alternative lengthening of telomerase pathway suggest that Atrx may have important functions in cellular transformation.

*ARID1A* and *ARID1B* are both members of the SWI/SNF transcriptional complex (Wang et al., 2004). SWI/SNF containing Arid1A was recently shown to be essential for neural crest development (Figure 1; Chandler and Magnuson, 2016). Homozygous loss of *Arid1A* results in embryonic lethality in mice, with mutant embryos succumbing to heart defects whereas heterozygous loss of *Arid1A* in the neural crest result in craniofacial defects in adult mice (Chandler and Magnuson, 2016). High frequency of *ARID1A* mutations have been reported in Coffin-Siris syndrome (CSS) a congenital disorder associated with intellectual disability or developmental delay, hypoplastic fifth fingernails, and patterning defects in the head and heart (Kosho et al., 2014). Like CSS, *ARID-1A* and *-1B* mutations are also found in a subset of neuroblastoma (Sausen et al., 2013). In addition, mutations in the histone acetyl transferase (HAC) genes *EP300* and *CREBBP*, the SWI2/SNF2 family member *TTF2* gene, the histone demethylase gene *KDM5A*, the chromatin remodeling zinc finger gene *IKZF1* and *ATRX*, described above, has also been reported in neuroblastoma (Sausen et al., 2013). SWI/SNF chromatin remodeling complexes are fundamental for coordinating a diverse set of chromatin alterations which influences the transcriptional output, DNA replication and repair (Tang et al., 2010). Genes encoding subunits of SWI/SNF complexes are mutated in over 20% of human cancers (St Pierre and Kadoch, 2017). The majority of these mutations of subunits within the SWI/SNF complexes results in loss of protein expression implicating SWI/SNF subunits as tumor suppressors (St Pierre and Kadoch, 2017). Although the effect *ARID1A/1B* mutations found in neuroblastoma need to be functionally analyzed, the fact that Arid-1A and -1B are part of the SWI/SNF tumor suppressor complexes suggest that these factors are important for deregulation of neural crest cells and neuroblastoma development.

### BARD1 as a Potential Factor for Neuroblastoma

*BRCA1* *Associated RING Domain 1 (BARD1)* encodes a protein which interacts with the N-terminal region of Brca1. Recent data indicates that different splice variants transcribed from the BARD1 gene can have either tumor suppressing or oncogenic functions. Full-length Bard1 proteins act as tumor suppressor with or without interaction with Brca1 whereas different splice variants detected in various cancers have oncogenic properties (Cimmino et al., 2017). As described above, GWAS identified SNPs within the *BARD1* locus that is strongly associated with neuroblastoma (Bosse and Maris, 2016). SNPs associated with high risk of neuroblastoma expressed high levels of *BARD1* splice variants whereas no correlation to neuroblastoma was detected in SNPs expressing full-length Bard1 (Capasso et al., 2013). One isoform, called Bard1β, has been shown to be important for neuroblastoma cell survival and to induce neoplastic transformation when overexpressed in murine fibroblasts (Bosse et al., 2012). The precise expression pattern and function of BARD1 and its splice variants during neural crest development has not yet been described. However, different copy number variants of the *BARD1* locus have been associated with congenital heart defects, aortic narrowing and tetralogy of Fallot all of which, as for neuroblastoma, have tissues that are derived from neural crest cells (Silversides et al., 2012). Through its interaction with Brca1, Bard1 has a pivotal role in DNA damage repair, ubiquitination, and transcriptional regulation to maintain genomic stability and deregulated expression of full-length Bard1 by SNPs splice variants located in the *BARD1* locus may have important implications for neuroblastoma development.

## Neuroblastoma as an Embryonal Tumor

Several lines of both clinical and experimental evidences suggest that neuroblastoma originates from dysregulation of cellular processes during early embryogenesis. As described in details above, gene mutations or amplifications as well as dysregulation of signal transduction pathways and epigenetic changes during neural crest development can induce preneoplastic lesions and eventually neuroblastoma. Studies done in Th-MYCN transgenic mice shows an accumulation of a population of small, blue round cells in the paravertebral ganglia from embryonic day 14. This population of cells called neuroblast hyperplasia later develops into neuroblastoma observed in 100% of the *MYCN* homozygous mice from postnatal week 6 (Hansford et al., 2004). Similar observations were also evident in the *Tg(dβh:EGFP-MYCN)* zebrafish model (Zhu et al., 2012). In humans the incidence of subclinical neuroblastomas is much higher than the frequency of clinical cases. Autopsies of sympathoadrenal tissues of infant whose cause of death were non-cancerous showed that the occurrence of precancerous neuroblasts was 40 times higher than the incidence of clinical disease (Beckwith and Perrin, 1963). Also, mass screening programs in Germany, France, Austria, North America and Japan during the 1990s for catecholamines in urine samples of infants found subclinical tumors at a frequency that was more than two-fold higher than the clinical incidence of neuroblastoma (Woods et al., 1996; Schilling et al., 2002). Although unsuccessful, these screening programs were initiated in order to prevent later metastatic neuroblastoma by early detection of localized tumors at infancy (Woods et al., 1996; Schilling et al., 2002).

There is one feature of neuroblastoma that is unique among human cancers and that is the propensity to spontaneously regress (Brodeur, 2018). Spontaneous regression is most evident in a specialized subgroup of infant neuroblastoma patients called 4S. Patients with stage 4S disease are infants up to 18 months of age and usually present with small abdominal primary tumors with metastases to the liver, skin, and bone marrow (Monclair et al., 2009). Stage 4S patients generally have very good prognosis and some undergo spontaneous regression without any treatment (D’Angio et al., 1971; Evans et al., 1971). The underlying mechanisms of the spontaneous tumor regression observed in stage 4S patients have not been defined but neurotrophin deprivation, loss of telomerase activity, epigenetic regulation or immune responses have been proposed to be responsible for neuroblastoma regression (Brodeur and Bagatell, 2014). Hence, regression of stage 4S neuroblastoma mimics features observed within the neural crest where excess neural precursors undergo apoptotic cell death at the final stage of sympathoadrenal maturation (Yuan and Yankner, 2000).

### Current Treatment of Neuroblastoma

Treatment of neuroblastoma patients diverges extensively between the different risk groups. Treatment of high-risk patients involves intense induction chemotherapy regimen that includes cisplatin, vincristine, carboplatin, etoposide and cyclophosphamide (COJEC; Pearson et al., 2008; Ladenstein et al., 2017). The induction therapy is followed by surgery and myeloablative therapy combined with reinfusion of hematopoietic stem cells and local radiotherapy. Maintenance treatment includes retinoic acid aiming to differentiate the remaining tumor cells and immunotherapy using anti-GD2 together with granulocyte macrophage colony stimulating factor (GM-CSF) and interleukin-2 (IL-2; Yu et al., 2010; Ladenstein et al., 2013; Park et al., 2014; Matthay et al., 2016; Berlanga et al., 2017; Shohet and Foster, 2017).

Patients categorized to the intermediate-risk group are given milder chemotherapy continued by surgical resection of the remaining tumor mass. Treatment of low-risk neuroblastomas include minimal chemotherapy and some children are cured by only surgery or no treatment caused by spontaneous tumor regression (Matthay et al., 2016; Berlanga et al., 2017; Shohet and Foster, 2017). Detailed descriptions of neuroblastoma treatment for different risk groups, and discussions of current clinical trials for neuroblastoma patients as well as investigational drugs depicting promising preclinical effects on neuroblastoma have recently been described by us and others (Matthay et al., 2016; Berlanga et al., 2017; Esposito et al., 2017; Moreno et al., 2017; Shohet and Foster, 2017; Whittle et al., 2017; Fletcher et al., 2018; Johnsen et al., 2018).

## Conclusion

Neuroblastoma can be regarded as neurodevelopmental disease originating from cells within the neural crest. As our detailed understanding of the complex network of inter- and intra-cellular communications within the neural crest are being resolved more knowledge of the cellular origin of neuroblastoma and mechanisms for neuroblastoma initiation will be understood. There is also a need to develop new preclinical models that mimic the genetic and chromosomal features seen in neuroblastoma. This has proven to be complicated since the majority of neuroblastoma present with very few gene mutations in which only a few have a proven oncogenic driver function. High-risk neuroblastomas usually have segmental chromosomal gain or losses. We currently have no knowledge on how these chromosomal aberrations arise within the pre-malignant cells or in neural crest-derived cells. These chromosomal gain or losses are demanding to study in preclinical *in vivo* models since the chromosomal make-up between mice and men are different. Neuroblastoma is still one of the most deadliest malignancies of childhood and we will need to refine current treatment protocols, include more targeted therapies, both pharmacologically and cellular which reflect the molecular landscape of individual tumors and to continue analyzing the influence of cells within the tumor microenvironment to search for novel therapy options. In order to accomplish this, we will need to develop more preclinical models that mimic the chromosomal and genetic landscape seen in neuroblastoma and to target these aberrations to specific cells within the neural crest.

## Author Contributions

JJ, CD and MW wrote the manuscript.

## Conflict of Interest Statement

The authors declare that the research was conducted in the absence of any commercial or financial relationships that could be construed as a potential conflict of interest.
